# Age-related variations in position and morphology of the temporomandibular joint in individuals with anterior openbite and crossbite: a multi-cross-sectional comparative study

**DOI:** 10.1186/s12903-022-02236-9

**Published:** 2022-05-23

**Authors:** Yanxi Chen, Jingxi Wang, Ying Li, Lingfeng Li, Nan Luo, Yan Wu, Hongwei Dai, Jianping Zhou

**Affiliations:** 1 Stomatological Hospital of Chongqing Medical University, 426 Songshi North Road, Chongqing, China; 2 Chongqing Key Laboratory of Oral Diseases and Biomedical Sciences, Chongqing, China; 3 Chongqing Municipal Key Laboratory of Oral Biomedical Engineering of Higher Education, Chongqing, China

**Keywords:** Temporomandibular joint, Cone-beam computed tomography, Anterior openbite, Anterior crossbite, Incisal guidance

## Abstract

**Background:**

This study aimed to compare the age-related positional and morphological characteristics of the temporomandibular joint (TMJ) between individuals with anterior openbite or crossbite and controls.

**Methods:**

This multi-cross-sectional comparative study analysed cone-beam computed tomography images of 750 participants, equally divided into the openbite, crossbite, and control groups (OBG, CBG, and CG, respectively). Each group was further divided into five subgroups (8–11 years, 12–15 years, 16–19 years, 20–24 years, and 25–30 years). Measurements of the TMJ included the position of the condyles in their respective fossae and morphology of the condyles and fossae. Data were submitted to statistical analysis. The study adhered to the STROBE Statement checklist for reporting of cross-sectional studies.

**Results:**

Condyles were positioned more posteriorly with increasing age in all groups, and the condylar position was more posterior in the OBG than in the CBG. The articular eminence inclination increased with age in all the groups. There were significant differences in the articular eminence inclination among the three major groups at the age of > 15 years, and the condylar path was flatter in the CBG than in the OBG.

**Conclusions:**

Age**-**related morphological and positional characteristics of the TMJ differed considerably among OBG, CBG and CG. Contrary to CBG, OBG was found to have relatively posterior condylar position and steeper condylar path.

**Supplementary Information:**

The online version contains supplementary material available at 10.1186/s12903-022-02236-9.

## Background

The temporomandibular joint (TMJ) with related anatomic structures is regarded as one of the most unique and essential components of the masticatory system, and its function is critical for maintaining proper occlusion and a stable stomatognathic system. It is well known that there is a significant correlation between the morphology of the TMJ and its function [1, 2]. Because the TMJ is capable of remodeling, morphologic and positional changes are thought to be an adaptive response to functional and mechanical requirements. Accumulating evidence indicates that various occlusal conditions cause functional adaptation in the neuromuscular system that is guided by proprioceptive feedback reflexes that originate in the teeth and this adaptation leads to structural and positional changes in the TMJ due to functional loads imposed on it [3].

Incisal guidance is among the occlusal factors and is essential for a harmonious and functional occlusion that can stabilise mandibular movement, reduce applied forces, and protect anatomical structures. Many researchers have emphasised that the incisal path must be coordinated with the condylar path and proposed that incisal guidance affects condylar guidance, which in turn influences condyle position and morphology [4, 5]. Interestingly, we previously reported that the relationships between the condyle and fossa are diverse in individuals with different incisor guiding angles, and incisal guidance is related to the growth and development of the TMJ [6]. Therefore, it is reasonable to suspect that no incisal guidance would affect the position and shape of the TMJ.

Individuals with anterior openbite or anterior crossbite have no incisal guidance, which has long-term effects on the masticatory movement. The aberrant movement of the lower jaw may put pressure on the TMJ, affecting its position and morphology. Abnormal external stress can readily result in TMJ structural degeneration in patients with limited compensating ability and slowed TMJ remodelling, generating detrimental effects on the TMJ and masticatory system. Some studies have revealed that patients with anterior openbite or crossbite have an increased risk of dysfunction of the TMJ, with more signs and symptoms such as muscle tiredness and orofacial pain [7–10]. Because condylar-fossa variation is strongly correlated with TMJ dysfunction [11, 12], altered TMJ position and morphology due to the lack of incisal path must be considered when planning orthodontic and prosthetic treatments in anterior openbite and crossbite cases.

To date, the relevant quantitative investigations remain insufficient. A few studies have investigated the TMJ configuration in individuals with anterior openbite or anterior crossbite. For example, Wohlberg et al*.* [13] concluded that anterior crossbite is associated with a reduced eminence height, causing a flatter condylar path; Koak et al*.* [14] suggested that the condyle inclination is smaller in openbite patients, and the function of the TMJ is more restricted due to poor incisal guidance. Notably, assessment of growth-related remodelling changes of the TMJ requires additional time. However, these aforementioned studies covered a small age range and involved fewer observations. In addition, age-related changes in the condyle and glenoid fossa can also affect TMJ and, if not taken into account, may lead to dysfunction [15, 16]. In general, there has been a lack of research on age-related variations in position and morphology of the TMJ in individuals with anterior openbite or those with anterior crossbite.

To our knowledge, despite a large amount of literature on the relationship between TMJ structures and different malocclusions, no study has compared age-related morphological and positional features of the TMJ between individuals with anterior openbite and those with anterior crossbite. Cone-beam computed tomography (CBCT) has recently been widely used to evaluate the TMJ region with a high level of reliability [17]. Hence, this study aimed to establish and compare normative and detailed data on the position and morphology of the TMJ among openbite, crossbite and unaffected individuals and to determine possible age-related adaptive changes in the TMJ using CBCT.

## Methods

### Sample-size calculation and sample selection

Ethical approval for this multi-cross-sectional comparative study was granted by the Stomatological Hospital of Chongqing Medical University Ethical Committee (No.2021-026). The sample size was calculated using an alpha value of 0.05 and a power of 90% based on a pilot experiment using PASS (Version 15.0, NCSS, LLC). A total of 750 participants participated in the study.

This retrospective study was conducted on individuals who had undergone CBCT examinations from February 2015 to July 2020 at the Stomatological Hospital of Chongqing Medical University. CBCT examinations were performed for several clinical purposes, e.g., orthodontic treatment, impacted teeth extraction, and third molar resolution. A total of 3785 untreated participants were screened for eligibility, and the criteria shown in Table 1 were used to select participants.

**Table 1:** Inclusion criteria for sample selection

Openbite group	Crossbite group	Control group
*Aged 8–30 years	*Aged 8–30 years	*Aged 8–30 years
*No vertical contact between upper and lower incisors at the maximum intercuspal position with overbite ≤ 0 mm	*All lower incisors locating on the labial side of the opposing upper teeth at the maximum intercuspal position	*All upper incisors locating on the labial side of the opposing lower teeth at the maximum intercuspal position with normal overjet and overbite
*No signs or symptoms of TMD^a^ based on recorded clinical data in the patients’ dental records	*No signs or symptoms of TMD^a^ based on recorded clinical data in the patients’ dental records	*No signs or symptoms of TMD^a^ based on recorded clinical data in the patients’ dental records
*No history of previous orthodontic treatment or restoration of anterior teeth	*No history of previous orthodontic treatment or restoration of anterior teeth	*No history of previous orthodontic treatment or restoration of anterior teeth
*No history of facial or dental trauma	*No history of facial or dental trauma	*No history of facial or dental trauma
*No transverse discrepancies with functional displacements	*No transverse discrepancies with functional displacements	*No transverse discrepancies with functional displacements
*No transverse skeletal discrepancies and no severe anteroposterior, vertical deformities	*No transverse skeletal discrepancies and no severe anteroposterior, vertical deformities	*No transverse skeletal discrepancies and no severe anteroposterior, vertical deformities
*No history of surgery in the craniofacial region	*No history of surgery in the craniofacial region	*No history of surgery in the craniofacial region
*No congenital craniofacial syndrome or anomaly (e.g., rheumatoid arthritis)	*No congenital craniofacial syndrome or anomaly (e.g., rheumatoid arthritis)	*No congenital craniofacial syndrome or anomaly (e.g., rheumatoid arthritis)
*No imaging evidence of condylar or glenoid fossa pathology (e.g., osteoarthritis, joint lesions and cysts, subcortical sclerosis and condylar hyperplasia)	*No imaging evidence of condylar or glenoid fossa pathology (e.g., osteoarthritis, joint lesions and cysts, subcortical sclerosis and condylar hyperplasia)	*No imaging evidence of condylar or glenoid fossa pathology (e.g., osteoarthritis, joint lesions and cysts, subcortical sclerosis and condylar hyperplasia)

TMD, temporomandibular disorder; TMJ, temporomandibular joint

^a^The Diagnostic Criteria for Temporomandibular Disorders was used during assessment of the TMD, which contains the muscular and/or TMJ pain, TMJ sounds and the range of the mandibular motions [18]

Following the application of inclusion and exclusion criteria, 750 non-TMD participants were selected and divided into three major groups (250 participants each): (1) openbite group (OBG) (81 males and 169 females; mean age: 18.18 ± 6.17 years), (2) crossbite group (CBG) (124 males and 126 females; mean age: 18.06 ± 6.90 years), and (3) control group (CG) (92 males and 158 females; mean age: 17.89 ± 5.66 years). Based on the characteristics of the TMJ’s growth and development, each major group was further divided by chronological age into five subgroups (50 participants each): groups I (8–11 years), II (12–15 years), III (16–19 years), IV (20–24 years), and V (25–30 years).

Right and left TMJs were examined separately, and a total of 1500 TMJ cases were measured and analysed.

### CBCT assessment and analysis

All CBCT (KaVo 3D exam, USA) images were obtained at the Stomatological Hospital of Chongqing Medical University (radiological parameters: 120 kV; 5 mA; voxel size, 0.4 mm; exposure time, 8.9 s; field of view, 16 × 17 cm). According to the imaging protocol, the Frankfort plane was adjusted to be parallel to the ground, and when the patient bit into maximum dental intercuspation, the CBCT scans were obtained. All images were obtained by a single radiologist.

**Fig. 1:**
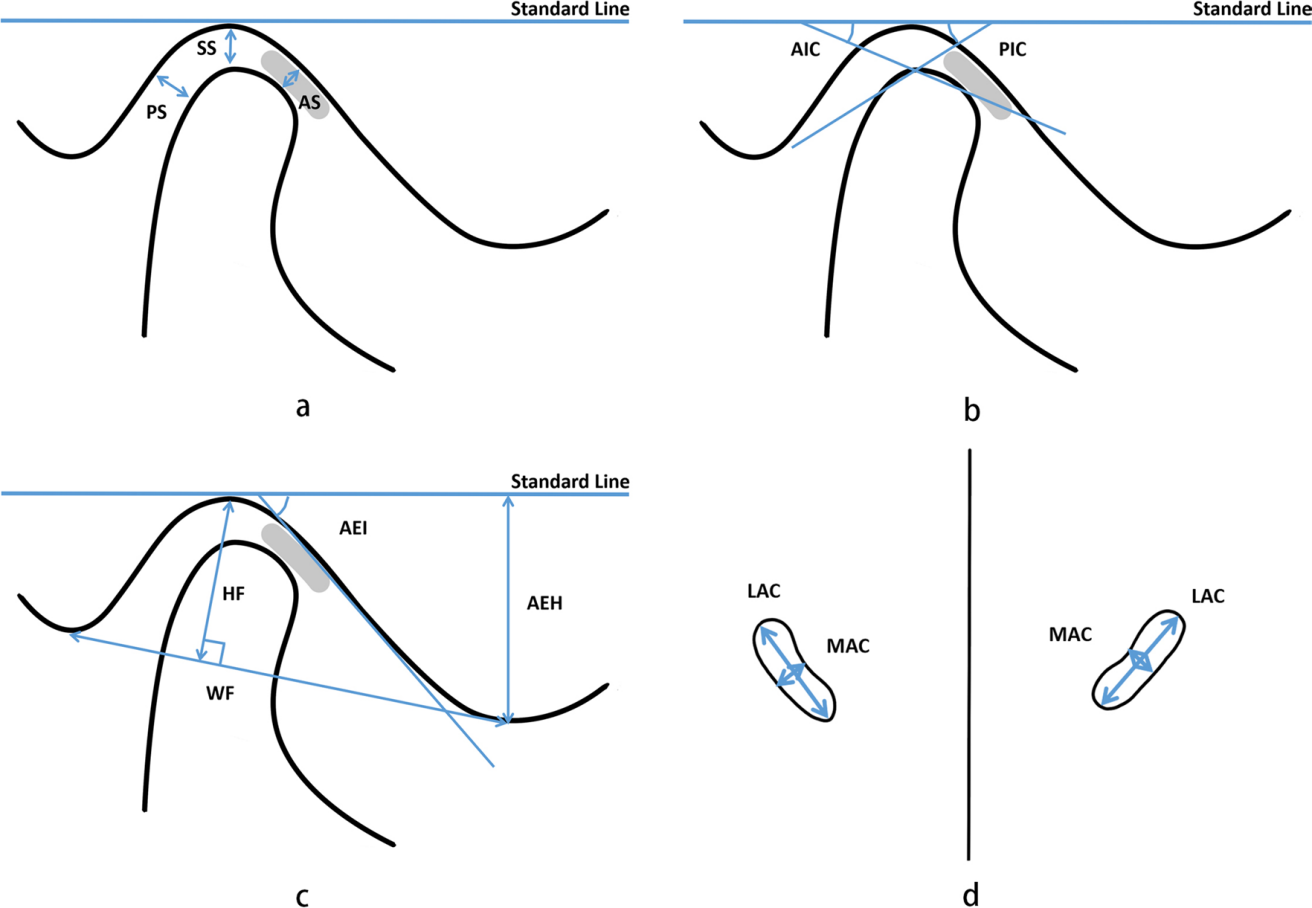
Measurements of the temporomandibular joints. **a** SS, superior space; AS, anterior space; PS, posterior space; **b** AIC, anterior inclination of the condyle; PIC, posterior inclination of the condyle; **c** HF, height of the fossa; WF, width of the fossa; AEH, articular eminence height; AEI, articular eminence inclination; **d** LAC, lateral inclination of the condyle; MAC, minor axis of the condyle

The CBCT data were analysed using the Dolphin11.9 software (Chatsworth, CA) by two investigators with experience in the evaluation of CBCT images. The Build X-Rays Tool was used to assess radiographically the anteroposterior and vertical skeletal characteristics of the participants by measuring the SNA, SNB, ANB, and FH-MP on constructed lateral images. The transverse skeletal characteristics were evaluated with the multiplanar CBCT images by measuring the maxillary basal width and the mandibular width, as described previously [19, 20]. Assessment of the TMJ position and morphology was performed in a manner similar to the studies by Chae et al*.* [21] and Ma et al*.* [22]. Initially, head-orientation images were standardised. When viewed from the front, the horizontal plane was parallel to the orbits. The skull was repositioned using the Frankfort horizontal plane, which was formed by the most superior point of the meatus acusticus externus on the right side and the most inferior point of the orbital rim on the left and right sides. To reconstruct the images of the sagittal and axial TMJ, the axial slice thickness was set to 0.5 mm to determine the largest and most pronounced condyle image on the left and right joints individually. Subsequently, the Standard Line (Fig. 1) was defined as a line tangent to the most superior point of the fossa and parallel to the Frankfort horizontal plane. Sagittal and axial measurements were performed based on the images as described as follows:

### Assessments of joint spaces (Fig. 1a).

1. Anterior joint space (AS), defined as the shortest distance between the most anterior point of the condyle and the posterior aspects of the articular tubercle;
2. Posterior joint space (PS), defined as the shortest distance between the most posterior point of the condyle and the posterior aspects of the mandibular fossa;
3. Superior joint space (SS), defined as the perpendicular distance between the most superior point of the condyle and the standard line.

### Assessments of condylar morphology (Fig. 1b, d)

1. Anterior inclination of the condyle (AIC), measured from the angle between the standard line and the line formed by the most superior point of the condyle to the point formed by the line drawn from the most superior point of the fossa tangent to the anterior aspects of the condylar head;
2. Posterior inclination of the condyle (PIC), measured from the angle between the standard line and the line formed by the most superior point of the condyle to the point formed by the line drawn from the most superior point of the fossa tangent to the posterior aspects of the condylar head;
3. Long axis of the condyle (LAC), the largest mediolateral diameter of the mandibular condylar processes;
4. Minor axis of the condyle (MAC), the largest anteroposterior diameter of the mandibular condylar processes.

### Assessment of fossa morphology (Fig. 1c).

1. Height of the fossa (HF), defined as the perpendicular distance between the most superior point of the fossa and the line formed by the most inferior point of the articular tubercle to the most inferior point of the postglenoid process;
2. Width of the fossa (WF), defined as the distance between the most inferior point of the articular tubercle and the most inferior point of the postglenoid process;
3. Articular eminence height (AEH), defined as the perpendicular distance between the most inferior point of the articular tubercle and standard line;
4. Articular eminence inclination (AEI), defined as the angle between the standard line and the best fit line on the posterior surface of the articular eminence.

Furthermore, according to Pullinger and Hollender [23], the anteroposterior condylar joint position (APCJP) was evaluated using the following formula: PS − AS/PS + AS × 100. A positive score indicates that the position is anterior, whereas a negative value indicates that the location is posterior.

### Reliability/reproducibility of the method

To ensure intra- and inter-examiner reliability, 50 randomly selected samples were analysed twice by two different observers within 20-day intervals. Intraclass correlation coefficients (ICCs) were used to assess the reliability and reproducibility of the measurements. Both results showed excellent test–retest reliability (ICC = 0.97–1.00), indicating that the reproducibility of the evaluation method was statistically acceptable.

### Statistical procedures

Data were analysed using the Statistical Package for the Social Sciences software, version 26 (IBM Corp, USA). The normality of data was confirmed using the Kolmogorov–Smirnov test, and all data were normally distributed. Quantitative data are presented as mean ± standard deviation (SD).

A paired t-test was performed to compare the left and right sides of the TMJ, and we found no statistical differences for any of the measurements (*P* > 0.1) (Additional file 1). The right and left TMJs were considered one unit, and the variables were averaged for subsequent analyses. The trend between age and TMJ characteristics was assessed using a linear trend test (P for trend). For intergroup comparisons, one-way analysis of variance (ANOVA) and Tukey’s post hoc test were used to determine statistically significant differences among various groups. A two-way multivariate ANOVA (two-way MANOVA) was performed to estimate the composite effect of age and occlusal characteristics of anterior teeth on the TMJ. Statistical significance was set at *P* < 0.05.

This study followed the STROBE statement for reports of observational studies (Additional file 2).

## Results

Table 2 shows the anteroposterior, vertical, and transverse skeletal features of the selected sample, with matched groups in the anteroposterior, vertical, and transverse relation to rule out the influence of these factors on the condyle-fossa relationship.

**Table 2:** The skeletal features of the study sample

Variables	Study group^a^			Control group			Study vs. Control (P value) ^b^		
	Class I (n = 280)	Class II (n = 98)	Class III (n = 122)	Class I (n = 144)	Class II (n = 42)	Class III (n = 64)	Class I	Class II	Class III
SNA (°)	81.89 ± 2.46	81.15 ± 1.71	82.16 ± 1.54	82.06 ± 2.34	80.87 ± 1.64	82.12 ± 1.58	0.190	0.189	0.873
SNB (°)	79.91 ± 2.37	76.65 ± 1.62	83.00 ± 1.59	80.09 ± 2.18	76.46 ± 1.67	83.02 ± 1.56	0.196	0.390	0.946
ANB (°)	1.98 ± 0.43	4.49 ± 0.18	-0.85 ± 0.21	1.97 ± 0.42	4.41 ± 0.38	-0.91 ± 0.19	0.760	0.161	0.127
FH-MP (°)	26.54 ± 4.47	26.70 ± 2.48	27.39 ± 3.71	26.64 ± 3.72	27.20 ± 2.80	27.38 ± 4.57	0.603	0.793	0.389
Maxillomandibular transverse difference	5.67 ± 0.60	5.65 ± 0.70	5.62 ± 0.58	5.69 ± 0.64	5.62 ± 0.58	5.53 ± 0.68	0.859	0.126	0.417

*P < .05

^a^Anterior openbite and crossbite subjects have been unified in the study group

^b^Paired t-test/Wilcoxon signed-rank test

### Joint spaces and condylar position

Linear trend tests suggested a trend toward increased mean SS with age in each major group (*P* < 0.005). Regarding the AS, statistically significant trends were observed in the three major groups (*P* = 0.001, *P* = 0.021, and *P* = 0.001, respectively). At the age of 12–15 years, the AS of the OBG was the largest, and that of the CG was the smallest. Additionally, changes of the PS in the CG (*P* = 0.002) and CBG (*P* = 0.021) with increasing age were statistically significant (Tables 3 and 4).

**Table 3:** Descriptive statistics and significant (*P*) values of analysis of variance (ANOVA) and Tukey’s tests for the temporomandibular joint measurements in all studied groups

	Control group (CG)	Openbite group (OBG)	Crossbite group (CBG)	ANOVA	Tukey’s test		
	Mean ± SD	Mean ± SD	Mean ± SD		CG-OBG	CG-CBG	OBG-CBG
*Superior space*							
I	2.438 ± 0.552	2.256 ± 0.612	2.444 ± 0.542	0.176	0.25	0.998	0.228
II	2.535 ± 0.649	2.639 ± 0.753	2.572 ± 0.648	0.744	0.729	0.961	0.877
III	2.711 ± 0.631	2.804 ± 0.851	2.574 ± 0.578	0.255	0.783	0.589	0.228
IV	2.754 ± 0.713	2.827 ± 0.723	2.730 ± 0.640	0.767	0.858	0.984	0.764
V	2.901 ± 0.635	2.919 ± 0.751	3.085 ± 0.737	0.362	0.991	0.399	0.473
*Anterior space*							
I	1.725 ± 0.435	1.816 ± 0.457	1.662 ± 0.309	0.165	0.502	0.718	0.143
II	1.822 ± 0.475	2.126 ± 0.678	1.837 ± 0.425	0.007**	0.015*	0.989	0.022*
III	2.041 ± 0.565	2.219 ± 0.700	1.995 ± 0.760	0.223	0.392	0.939	0.229
IV	1.892 ± 0.454	2.006 ± 0.582	1.981 ± 0.670	0.583	0.584	0.72	0.974
V	2.065 ± 0.629	2.186 ± 0.538	2.002 ± 0.556	0.27	0.546	0.848	0.25
*Posterior space*							
I	2.029 ± 0.550	2.104 ± 0.561	2.139 ± 0.592	0.614	0.787	0.598	0.949
II	1.906 ± 0.554	2.051 ± 0.602	2.115 ± 0.486	0.154	0.387	0.142	0.83
III	2.028 ± 0.530	2.109 ± 0.536	1.983 ± 0.535	0.614	0.729	0.907	0.467
IV	1.682 ± 0.333	1.866 ± 0.497	1.944 ± 0.392	0.006**	0.07	0.005**	0.613
V	1.801 ± 0.363	2.010 ± 0.559	1.971 ± 0.398	0.049*	0.055	0.143	0.901
*Anteroposterior condylar joint position*							
I	0.079 ± 0.159	0.072 ± 0.181	0.114 ± 0.150	0.399	0.979	0.536	0.419
II	0.021 ± 0.135	-0.014 ± 0.205	0.069 ± 0.132	0.039*	0.525	0.303	0.030*
III	0.000 ± 0.163	-0.019 ± 0.195	0.010 ± 0.210	0.748	0.879	0.961	0.732
IV	-0.033 ± 0.180	-0.033 ± 0.173	0.007 ± 0.176	0.428	1.000	0.497	0.496
V	-0.038 ± 0.202	-0.042 ± 0.178	0.001 ± 0.175	0.445	0.993	0.551	0.48
*Anterior inclination of the condyle*							
I	27.265 ± 4.933	27.203 ± 5.336	25.454 ± 5.713	0.16	0.998	0.21	0.233
II	29.539 ± 5.041	29.032 ± 6.226	25.163 ± 5.236	0.000***	0.891	0.000***	0.002**
III	31.414 ± 5.929	29.042 ± 6.913	27.069 ± 7.056	0.006**	0.179	0.004**	0.302
IV	31.503 ± 6.537	30.349 ± 7.133	27.070 ± 8.128	0.008**	0.709	0.008**	0.067
V	31.743 ± 5.732	30.409 ± 6.764	28.835 ± 6.466	0.075	0.545	0.06	0.43
*Posterior inclination of the condyle*							
I	27.911 ± 5.818	27.501 ± 6.023	28.681 ± 6.801	0.631	0.942	0.81	0.611
II	27.331 ± 4.719	26.886 ± 6.202	25.861 ± 4.961	0.371	0.909	0.355	0.603
III	27.536 ± 4.944	27.197 ± 8.037	29.701 ± 7.547	0.154	0.968	0.27	0.175
IV	26.832 ± 4.092	25.893 ± 6.127	27.784 ± 6.045	0.232	0.671	0.663	0.202
V	28.007 ± 3.638	27.437 ± 5.620	28.043 ± 6.190	0.812	0.851	0.999	0.833
*Long axis of the condyle*							
I	16.811 ± 1.792	16.668 ± 1.672	16.887 ± 1.617	.807	.907	.973	.795
II	17.850 ± 1.723	16.731 ± 1.642	17.925 ± 1.742	0.001**	0.004**	0.974	0.002**
III	18.523 ± 2.354	18.013 ± 2.246	18.826 ± 2.091	0.188	0.490	0.776	0.166
IV	19.038 ± 2.110	18.044 ± 2.407	19.396 ± 2.700	0.017*	0.103	0.740	0.016*
V	19.616 ± 3.648	18.092 ± 2.824	20.016 ± 2.342	0.004**	0.031*	0.782	0.004**
*Minor axis of the condyle*							
I	8.974 ± 1.019	8.781 ± 0.880	8.533 ± 0.744	0.048*	0.524	0.037*	0.345
II	9.224 ± 0.963	8.864 ± 0.798	8.978 ± 0.919	0.125	0.114	0.358	0.801
III	9.147 ± 1.008	9.026 ± 1.274	8.981 ± 0.859	0.721	0.836	0.714	0.976
IV	9.508 ± 0.868	9.029 ± 1.189	9.416 ± 1.688	0.136	0.145	0.933	0.278
V	9.125 ± 0.860	9.255 ± 1.310	9.529 ± 0.948	0.153	0.812	0.139	0.400
*Height of the fossa*							
I	6.179 ± 0.686	6.168 ± 1.055	5.922 ± 0.895	0.268	0.998	0.322	0.354
II	6.509 ± 0.901	6.502 ± 1.619	6.251 ± 0.922	0.471	1.000	0.528	0.546
III	6.808 ± 0.785	6.746 ± 1.335	6.622 ± 0.978	0.67	0.954	0.654	0.828
IV	6.867 ± 1.137	6.489 ± 1.290	6.484 ± 0.845	0.143	0.205	0.197	1.000
V	6.464 ± 1.168	6.206 ± 1.367	6.197 ± 0.884	0.426	0.506	0.482	0.999
*Width of the fossa*							
I	17.518 ± 1.404	18.333 ± 1.983	18.896 ± 2.104	0.001**	0.075	0.001**	0.286
II	18.146 ± 2.099	18.755 ± 1.451	19.403 ± 2.054	0.005**	0.245	0.003**	0.204
III	18.605 ± 1.748	19.114 ± 1.765	19.597 ± 1.666	0.018*	0.307	0.013*	0.344
IV	18.505 ± 1.615	18.995 ± 2.015	19.006 ± 2.124	0.336	0.415	0.399	1.000
V	17.168 ± 1.818	18.461 ± 1.441	18.557 ± 1.646	0.000***	0.000***	0.000***	0.954
*Articular eminence height*							
I	6.176 ± 0.930	6.148 ± 1.452	5.211 ± 0.910	0.000***	0.992	0.000***	0.000***
II	6.532 ± 1.603	6.496 ± 1.407	5.617 ± 1.156	0.001**	0.991	0.004**	0.006**
III	6.607 ± 1.207	6.562 ± 1.422	5.620 ± 1.276	0.000***	0.984	0.001**	0.001**
IV	6.798 ± 1.315	6.569 ± 1.581	5.772 ± 1.338	0.001**	0.698	0.001**	0.015**
V	6.815 ± 1.538	6.654 ± 1.278	6.122 ± 1.067	0.024*	0.812	0.024*	0.108
*Articular eminence inclination*							
I	47.117 ± 9.726	46.773 ± 11.963	44.553 ± 9.038	0.405	0.985	0.43	0.531
II	51.070 ± 11.211	50.005 ± 9.495	46.909 ± 9.722	0.108	0.86	0.105	0.284
III	52.970 ± 11.167	50.047 ± 12.263	47.002 ± 9.960	0.031*	0.393	0.023*	0.363
IV	54.960 ± 10.569	51.524 ± 12.117	47.113 ± 10.338	0.002**	0.268	0.001**	0.116
V	56.678 ± 10.552	52.210 ± 11.656	48.788 ± 9.581	0.001**	0.093	0.001**	0.245

I, 8–11 years; II, 12–15 years; III, 16–19 years; IV, 20–24 years; V, 25–30 years; ANOVA, analysis of variance; SD, standard deviation

*P < .05; **P < .01; ***P < .001

**Table 4:** Linear trend tests in the three major groups

Variables	Control group		Openbite group		Crossbite group	
	F	P	F	P	F	P
Superior space	16.106	0.000***	20.817	0.000***	25.913	0.000***
Anterior space	10.53	0.001**	5.377	0.021*	10.532	0.001**
Posterior space	10.195	0.002**	2.282	0.132	5.423	0.021*
Anteroposterior condylar joint position	14.37	0.000***	8.853	0.003**	14.222	0.000***
Anterior inclination of the condyle	18.575	0.000***	7.056	0.008**	8.63	0.004**
Posterior inclination of the condyle	0.021	0.884	0.151	0.698	0.052	0.82
Long axis of the condyle	39.191	0.000***	17.810	0.000***	65.509	0.000***
Minor axis of the condyle	1.919	0.167	4.937	0.027*	25.086	0.000***
Height of the fossa	4.727	0.031*	0.011	0.917	3.736	0.054
Width of the fossa	0.189	0.664	0.402	0.526	1.55	0.214
Articular eminence height	6.632	0.011*	2.872	0.091	14.533	0.000***
Articular eminence inclination	23.305	0.000***	5.763	0.017*	3.968	0.047*

F, F statistics; P, *P-*value

*P < .05; **P < .01; ***P < .001

**Fig. 2:**
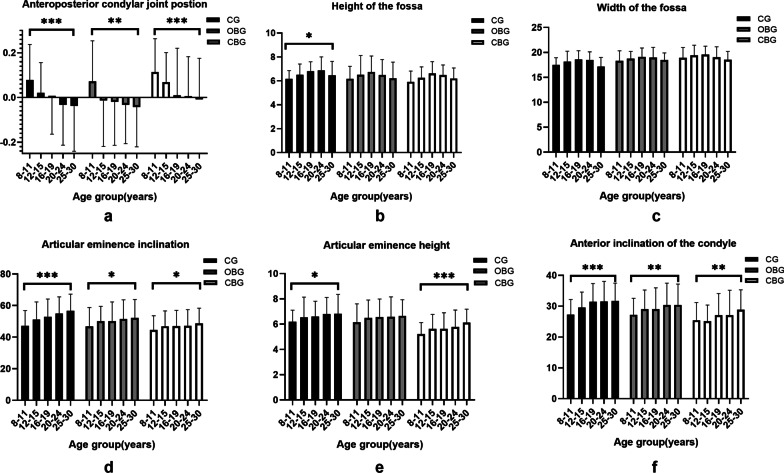
Box-and-whisker plots showing the temporomandibular joint measurements of different age subgroups. Significant differences were defined by the means of the linear trend test (*P < .05. **P < .01. ***P < .001). **a** APCJP, anteroposterior condylar joint position; **b** HF, height of the fossa; **c** WF, width of the fossa; **d** AEI, articular eminence inclination; **e** AEH, articular eminence height; **f** AIC, anterior inclination of the condyle

The APCJP decreased with increasing age, and the condyles of all three major groups were positioned significantly more posteriorly (*P* < 0.001, *P* = 0.003, and *P* < 0.001, respectively). Moreover, the condyle position was mainly anterior and concentric in the CBG and was relatively posterior in the OBG (Fig. 2a; Tables 3 and 4).

### Condylar morphology

The AIC significantly increased with increasing age in the three main groups (*P* < 0.001, *P* = 0.008, and *P* = 0.004, respectively). ANOVA indicated significant differences in the AIC among the three major groups, and the AIC of the OBG was smaller than that of the CG but greater than that of the CBG at the ages of 12–15 (*P* < 0.001), 16–19 (*P* = 0.006), and 20–24 (*P* = 0.008) years (Fig. 2f; Tables 3 and 4). However, there were no statistically significant trends in the PIC with age in each major group (Tables 3 and 4).

With the increase in age, the LAC increased (from 8–11 years to 25–30 years) (*P* < 0.001) and was significantly longer in the CBG than in the CG and OBG at the ages of 12–15 (*P* = 0.001), 20–24 (*P* = 0.017), and 25–30 (*P* = 0.004) years. The MAC tended to increase with age in the OBG (*P* = 0.027) and CBG (*P* < 0.001). The only significant MAC difference was detected among three major groups at 8–11 years of age (*P* = 0.048) (Tables 3 and 4).

### Fossa morphology

The average HF increased first and then decreased with age in the CG (*P* = 0.031), OBG (*P* = 0.917), and CBG (*P* = 0.054). The turning point of the CG (20–24 years) was different from that of the other two major groups (16–19 years). No significant differences were found among the three major groups (Fig. 2b; Tables 3 and 4).

The WF tended to increase from 8–11 to 16–19 years, and then decreased with increasing age in all major groups. Significant differences were observed in WF among the three major groups at the ages of 8–11 (*P* = 0.001), 12–15 (*P* = 0.005), 16–19 (*P* = 0.018), and 25–30 (*P* < 0.001) years (Fig. 2c; Tables 3 and 4).

The AEH increased with age in all three major groups, but the trends were statistically significant only in the CG (*P* = 0.011) and CBG (*P* < 0.001). In the same age groups, the AEH of the OBG was smaller than that of the CG but greater than that of the CBG (Fig. 2e; Tables 3 and 4).

The AEI tended to increase with age in the OBG (*P* = 0.017), CBG (*P* = 0.047), and CG (*P* < 0.001). The AEI was flatter in the CBG than in the CG and OBG at the ages of 16–19(*P* = 0.031), 20–24(*P* = 0.002) and 25–30 (*P* = 0.001) years (Fig. 2d; Tables 3 and 4).

### Effects of age or/and occlusal characteristics of anterior teeth on TMJ

Two-way MANOVA (Table 5) showed that age and occlusal characteristics of anterior teeth significantly affected the TMJ. However, no composite effect was observed in any of the measurements.

**Table 5:** Results of two-way multivariate analysis of variance for the temporomandibular joint measurements

Variables	Age		Occlusal characteristics of anterior teeth		Interaction with Age and Occlusal characteristics of anterior teeth	
	F	P	F	P	F	P
Superior space	15.874	0.000***	0.063	0.939	1.101	0.36
Anterior space	9.861	0.000***	7.524	0.001**	0.562	0.809
Posterior space	6.268	0.000***	6.376	0.002**	0.804	0.599
Anteroposterior condylar joint position	10.552	0.000***	4.796	0.009**	0.223	0.987
Anterior inclination of the condyle	8.219	0.000***	21.351	0.000***	0.679	0.711
Posterior inclination of the condyle	2.046	0.086	1.912	0.148	0.881	0.532
Long axis of the condyle	29.475	0.000***	16.370	0.000***	1.171	0.314
Minor axis of the condyle	7.115	0.000***	2.424	0.089	1.666	0.103
Height of the fossa	8.153	0.000***	3.871	0.021*	0.334	0.953
Width of the fossa	8.578	0.000***	24.092	0.000***	0.844	0.564
Articular eminence height	5.651	0.000***	36.585	0.000***	0.262	0.978
Articular eminence inclination	7.626	0.000***	17.856	0.000***	0.655	0.731

F, F statistics; P, *P-*value

*P < .05; **P < .01; ***P < .001

## Discussion

The TMJ position and morphology varies among individuals and can be altered by a variety of factors, including the functional pressures exerted on it. This could be attributed to the close link between shape and function, which varies depending on occlusal characteristics [24]. Incisal guidance is one of the occlusal factors which can stabilise the movement of the condyle and maintain the health of the oral and craniofacial structures [25]. Therefore, abnormal movement of the lower jaw without incisor guidance may affect the shape, position, and function of the TMJs and masticatory system over time. Thus, understanding of the condyle-fossa relationships in anterior openbite and crossbite (no incisal guidance) could provide insights into the pathological effects of no incisal guidance on the TMJ. To the best of our knowledge, this study is the first to evaluate the TMJ characteristics in different age groups with anterior openbite or crossbite compared with their unaffected peers.

Investigating the effect of anterior openbite and crossbite on the position and shape of the TMJ anatomy was the primary objective of this study. Additionally, developmental and adaptive changes of the TMJ are age-related. The growth and development of the mandibular condyle are strongly linked to the growth and development of the mandible. It has been reported that there are two periods of increased growth of the TMJ: between the ages of 5 and 10 years and between the ages of 10 and 15, with the craniofacial growth stopping around the age of 20 [26, 27]. Katsavrias et al. [28] reported that the articular eminence height increases rapidly until the age of 7 years, then slows until the age of 11 years, regaining its full height by the age of 20 years. Sülün et al. [29] reported that the eminence inclination in healthy patients peaks between the ages of 21 and 30 years. Based on the TMJ’s growth and development characteristics, subjects were divided into five age groups to explore the age-related changes in the condyle and glenoid fossa, which was the second objective of this study.

In the present study, the condyles were more posteriorly positioned with age (*P* < 0.05); this finding aligns with the results obtained by Cohlmia et al*.* [30], indicating that the condyle-glenoid fossa relationship changes with increasing age. However, Liu et al*.* [31] failed to find a changing tendency in condylar position with increasing age, probably due to different age ranges in various age groups.

Moreover, we found that the condyle position was mainly anterior and concentric in the anterior crossbite. This finding contradicts that of Cohlmia et al*.* [30]*,* who found no significant differences between patients with and without anterior crossbites in the condylar position. This discrepancy potentially results from the different inclusion criteria since patients with other types of malocclusions were also included in their study. Additionally, we observed that the position of the condyle in openbite was relatively posterior. The condyle position refers to the relative position of the condylar in the mandibular fossa in concentric occlusion and is the final product of many dynamic changes, which are related to growth, remodelling, and response to functional changes and so on [32, 33]. Different occlusal circumstances lead to different stress distributions in the TMJ, as described previously [34]. Thus, the position of the TMJ, which is affected by the pressures load on it, varies. The position of the lower incisors locating on the labial side of the opposing upper teeth, as an occlusal characteristic of the anterior crossbite, affects the mandibular movement guided by neuromuscular reflexes, which possibly changes the position of the condylar for functional adaptation. Furthermore, in anterior openbite individuals, no contact between the upper and lower teeth in the vertical direction might also influence the motion trajectory of the mandible; thus, the condyle position is relatively backward. To sum up, the position of the TMJ is subjected to a variety of functional and mechanical stresses during oral function, depending on the subject’ occlusal characteristics. Additionally, the sagittal position of the condyle is associated with TMJ dysfunction because the posterior position of the condyle may exert pressure on the retrodiscal tissues and sensory nerves, leading to TMD [35]. The higher incidence of the posterior condylar position suggests the anatomical preponderance of TMD and disk instability in anterior openbite; this finding is in line with the increased prevalence of clinical signs and symptoms of TMD in anterior openbite [9, 36].

Regarding the HF and WF, the trends of these data first increased and then decreased with increasing age in the three major groups, except that the turning point of the HF in the CG was different from that in the other two major groups. Different turning points might have been caused by the incisor guidance; the early increase may be the result of growth and development, while the late decrease is viewed as an adaptive response due to functional and mechanical constraints [6]. Except for the largest fossa width, the HF was lower in the CBG than in the other two groups; consistently, the AEH was lowest in the CBG. These results suggest that individuals with anterior crossbite have relatively shallow and wide glenoid fossa.

The orbit of the condyle, which moves out of the most superior and anterior position from the glenoid fossa, is guided by the posterior wall of the articular eminence and is commonly termed ‘condylar guidance’. Hence, the AEH and AEI influence the condylar path. In our study, AEH and AEI showed a sustained increase with ageing in each major group, and the AIC also increased with ageing; the findings are consistent with previous results [6, 37, 38]. Due to the coordination between AIC, AEI, and AEH in anatomical structure and jaw movement, their changing trends with age are similar. Moreover, some researchers [39] demonstrated that steeper articular eminences forced posterior disk rotation more prominently because of larger vertical movement upon opening as the condyle shifts anteriorly. Therefore, considering the peak age of TMD involvement, the steeper inclination of articular eminences at the ages of 20–30 years could be an anatomical aetiology. Because the aetiological and pathophysiological factors of TMD are complicated, factors such as psychoemotional stress may potentially influence the muscular endurance and masticatory system, consequently affecting the shape and position of the TMJ. Consistent with it, Wieckiewicz et al*.* [40] assessed the prevalence of TMD among Polish university students and demonstrated that emotional burden is a risk factor for muscular disorders which can impair the adaptive capacity of the masticatory system and may lead to a greater risk of TMD.

This study indicated significant differences in AEI among the three major groups at the age of > 15 years. Moreover, we found that the AEI of the CBG was significantly smaller than that of the CG, probably due to the flattening of the condylar path in the CBG; this finding is consistent with that of Wohlberg et al*.* [13]. The main reason for these results may be the occlusal characteristics in the CBG, which plays a role in the mastication pattern controlled by neuromuscular elements. The neuromuscular reflex changes the contractility of the masticatory muscles, thereby affecting the stress of the TMJ, which is the underlying cause of joint remodelling. Although no statistically significant differences were found between the CG and OBG, the condylar inclination of the OBG was flatter than that of the CG. This result agrees with those of previous studies regarding mandibular movement in patients with openbite and could be explained by the limitations of the effect of incisal guidance, as described by Koak et al*.* [14]. Moreover, the condylar path in the OBG was steeper than that in the CBG, suggesting that masticatory function in anterior openbite participants differed from that in anterior crossbite participants and mechanical force loaded on the TMJ was thus altered, thereby causing a difference in the condylar path. Consistently, Thorsten et al*.* [41] demonstrated that functional demands influence jaw muscles, which are able to modify their anatomical features, such as dimensions, cross-sectional area, and fibre phenotype. Taken together, the condylar path is affected by the incisal guidance; this result is in accordance with the conclusion of Hickey et al*.* [42] that incisal guidance has some influence on condylar guidance.

Accumulating evidence indicates that the condyle-fossa relationship is related to a variety of factors, such as age [15, 16], sex [21, 30] and occlusal factors (e.g., incisal guidance, tooth loss, and dental abrasion) [6, 38, 43]. Our results, similar to others, indicated that age and occlusal features of anterior teeth influenced the TMJ characteristics, but no composite effect of both was found. However, whether interactions between other correlative factors associated with TMJ exist and how they interact with each other should be further investigated in future studies considering the lack of previous related reports.

Functions and morphology are closely linked. It is widely recognized that pressure originating from the contractility of the masticatory muscles during chewing movements can affect the TMJ. This relationship implies that the position and morphology of the TMJ are somewhat determined by the forces pressing on it. Maeda et al*.* [44] suggested that the proliferation and matrix synthesis of condylar chondrocytes would be influenced by the pressure exerting on the TMJ, which is consistent with this assumption. Various occlusal conditions result in functional adaptation in the neuromuscular system which is guided by proprioceptive feedback reflexes that originate in the teeth [3]. This adaptation, in turn, influences the jaw muscles and causes structural and positional changes in the TMJ due to functional loads imposed on it. Thus, the TMJ loading differs between persons with specific occlusal conditions and those having other dentofacial morphologies, as does mechanical stress on the TMJ during oral function [34, 45, 46]. Taken together, incisor guidance has important implications for mandibular movement. The movement of the condyle, muscular contraction, neuromuscular health, TMJ, and associated structures in individuals with anterior openbite and crossbite will be disturbed without incisal guidance [47, 48], thus changing the condyle-fossa relationships, which the results of this study can support.

This study has some limitations. Longitudinal data could better explain the relationship between the shape and position of the TMJ and incisor guidance than multi-cross-sectional data. In addition, more detailed analyses of clinical data, such as muscle myoelectric activity and biting force, are required for further evaluation.

## Conclusions

This study compared age-related variations in the position and morphology of the TMJ among the control, openbite, and crossbite groups. We found significant differences in condyle-fossa relationships and trends with age among the three groups. Contrary to the crossbite groups, individuals with anterior openbite were found to have relatively posterior condylar position and steeper condylar path. These findings can be clinically useful when evaluating the position and morphology of the TMJ with CBCT images in individuals with anterior openbite and crossbite.

## Supplementary Information

**Additional file 1**. Side comparison of the measurements among three major groups.

**Additional file 2**. STROBE Statement—checklist of items that should be included in reports of observational studies.

## Data Availability

The data that support the findings of this study are available from the Stomatological Hospital of Chongqing Medical University, but restrictions apply to the availability of these data, which were used under license for the current study, and so are not publicly available. Data are however available from the authors upon reasonable request and with permission of the Stomatological Hospital of Chongqing Medical University.

## Abbreviations

AEH: Articular eminence height
AEI: Articular eminence inclination
AIC: Anterior inclination of the condyle
ANOVA: Analysis of variance
APCJP: Anteroposterior condylar joint position
AS: Anterior joint space
CBCT: Cone-beam computed tomography
CBG: Crossbite group
CG: Control group
HF: Height of the fossa
ICCs: Intraclass correlation coefficients
LAC: Long axis of the condyle
MAC: Minor axis of the condyle
OBG: Openbite group
PIC: Posterior inclination of the condyle
PS: Posterior joint space
SD: Standard deviation
SS: Superior joint space
TMJ: Temporomandibular joint
TMD: Temporomandibular disorders
WF: Width of the fossa
