# Supplementary material for: Age-related variations in position and morphology of the temporomandibular joint in individuals with anterior openbite and crossbite: a multi-cross-sectional comparative study
Source: BMC Oral Health. 2022 May 23;22:200. doi: 10.1186/s12903-022-02236-9 (PMC9125888; doi:10.1186/s12903-022-02236-9)
Supplement: Supplementary file 1 — Additional file 1. Side comparison of the measurements among three major groups. [file 12903_2022_2236_MOESM1_ESM.docx]

|  |  |  |  |  |  |  |  |  |  |
| --- | --- | --- | --- | --- | --- | --- | --- | --- | --- |
|  | CG | | | OBG | | | CBG | | |
|  | Right side | Left Side |  | Right side | Left Side |  | Right side | Left Side |  |
| Measurements | Mean±SD | Mean±SD | P-Value | Mean±SD | Mean±SD | P-Value | Mean±SD | Mean±SD | P-Value |
| Superior space | 2.68±0.73 | 2.65±0.69 | 0.397 | 2.69±0.87 | 2.69±0.87 | 0.969 | 2.69±0.78 | 2.67±0.81 | 0.663 |
| Anterior space | 1.93±0.65 | 1.88±0.57 | 0.184 | 2.08±0.70 | 2.06±0.68 | 0.679 | 1.87±0.64 | 1.92±0.68 | 0.195 |
| Posterior space | 1.89±0.55 | 1.89±0.58 | 0.900 | 2.02±0.67 | 2.04±0.68 | 0.621 | 2.00±0.58 | 2.06±0.63 | 0.152 |
| Anteroposterior condylar joint position | -0.01±0.20 | 0.00±0.19 | 0.501 | -0.01±0.22 | -0.01±0.22 | 0.559 | 0.04±0.19 | 0.04±0.22 | 0.759 |
| Anterior inclination of the condyle | 30.41±8.27 | 30.18±6.90 | 0.709 | 29.38±8.41 | 29.04±7.70 | 0.564 | 26.36±8.16 | 26.72±7.95 | 0.501 |
| Posterior inclination of the condyle | 27.07±6.94 | 27.98±7.34 | 0.180 | 27.17±10.09 | 26.84±8.88 | 0.708 | 28.59±8.54 | 27.49±8.87 | 0.138 |
| Height of the fossa | 6.56±1.20 | 6.57±1.10 | 0.971 | 6.45±1.40 | 6.40±1.47 | 0.461 | 6.32±1.31 | 6.27±1.34 | 0.649 |
| Width of the fossa | 17.99±1.93 | 17.98±2.11 | 0.936 | 18.69±1.97 | 18.77±1.92 | 0.473 | 19.01±2.52 | 19.28±2.75 | 0.437 |
| Articular eminence height | 6.64±1.48 | 6.53±1.72 | 0.321 | 6.42±1.62 | 6.55±1.79 | 0.296 | 5.63±1.26 | 5.71±1.36 | 0.291 |
| Articular eminence inclination | 52.69±11.97 | 52.43±12.27 | 0.676 | 49.65±12.39 | 50.57±13.07 | 0.167 | 46.36±11.08 | 47.39±12.15 | 0.200 |
| Long axis of the condyle | 18.32±3.49 | 18.42±2.40 | 0.612 | 17.50±2.49 | 17.52±2.46 | 0.804 | 18.61±2.55 | 18.61±2.55 | 0.983 |
| Minor axis of the condyle | 9.16±1.06 | 9.23±1.04 | 0.216 | 8.94±1.12 | 9.04±1.28 | 0.101 | 9.13±1.34 | 9.05±1.19 | 0.292 |

**Additional File 1:** Side comparison of the measurements among three major groups.

Abbreviations: SD, standard deviation.

*P<.05. **P<.01. ***P<.001.
