# Supplementary material for: Age-related variations in position and morphology of the temporomandibular joint in individuals with anterior openbite and crossbite: a multi-cross-sectional comparative study
Source: BMC Oral Health. 2022 May 23;22:200. doi: 10.1186/s12903-022-02236-9 (PMC9125888; doi:10.1186/s12903-022-02236-9)
Supplement: Supplementary file 2 — Additional file 2. STROBE Statement—checklist of items that should be included in reports of observational studies. [file 12903_2022_2236_MOESM2_ESM.docx]

STROBE Statement—checklist of items that should be included in reports of observational studies

|  | **Item No.** | **Recommendation** | **Page No.** | **Relevant text from manuscript** |
| --- | --- | --- | --- | --- |
| **Title and abstract** | 1 | (*a*) Indicate the study’s design with a commonly used term in the title or the abstract | 1 | a multi-cross-sectional study |
|  |  | (*b*) Provide in the abstract an informative and balanced summary of what was done and what was found | 2 | to compare the age-related positional and morphological characteristics of the temporomandibular joint (TMJ) between individuals with anterior openbite or crossbite and controls  Age-related morphological and positional characteristics of the TMJ were considerably differed among OBG, CBG and CG.  . |
| **Introduction** |  |  |  |  |
| Background/rationale | 2 | Explain the scientific background and rationale for the investigation being reported | 3,4 | No incisor guidance would affect the position and shape of the TMJ. |
| Objectives | 3 | State specific objectives, including any prespecified hypotheses | 5 | to establish and compare normative and detailed data on the position and morphology of the TMJ among openbite, crossbite and unaffected individuals and to determine possible age-related adaptive changes in the TMJ using CBCT. |
| **Methods** |  |  |  |  |
| Study design | 4 | Present key elements of study design early in the paper | 5 | This was a multi-cross-sectional observational study |
| Setting | 5 | Describe the setting, locations, and relevant dates, including periods of recruitment, exposure,  follow-up, and data collection | 5 | Conducted at the Stomatological Hospital of Chongqing Medical University, China |
| Participants | 6 | (*a*) *Cohort study*—Give the eligibility criteria, and the sources and methods of selection of participants. Describe methods of follow-up  *Case-control study*—Give the eligibility criteria, and the sources and methods of case ascertainment and control selection. Give the rationale for the choice of cases and controls *Cross-sectional study*—Give the eligibility criteria, and the sources and methods of selection of  participants | Table 1 | Conducted on individuals’ CBCT examinations and dental records from February 2015 to July 2020 |
|  |  | (*b*) *Cohort study*—For matched studies, give matching criteria and number of exposed and unexposed  *Case-control study*—For matched studies, give matching criteria and the number of controls per  case |  | Not applicable |
| Variables | 7 | Clearly define all outcomes, exposures, predictors, potential confounders, and effect modifiers.  Give diagnostic criteria, if applicable | 8-10 | The primary outcomes were values of TMJ position and morphology in individuals with anterior openbite, crossbite and controls,which were determined using CBCT |
| Data sources/  measurement | 8* | For each variable of interest, give sources of data and details of methods of assessment  (measurement). Describe comparability of assessment methods if there is more than one group | Figure 1 |  |
| Bias | 9 | Describe any efforts to address potential sources of bias | Table 2 | Table 2 showed the anteroposterior, vertical, and transverse skeletal features of the selected sample, with matched groups in the anteroposterior, vertical, and transverse relation to rule out the influence of these factors on the condyle- fossa relationship.  To ensure intra- and inter-examiner reliability, 50 randomly selected samples were analysed twice by two different observers within 20-day intervals. |
| Study size | 10 | Explain how the study size was arrived at | 5 | The sample size was calculated using an alpha value of 0.05 and a power of 90% based on a pilot experiment using PASS (Version 15.0, NCSS, LLC). A total of 750 participants participated in the study. |
| Quantitative variables | 11 | Explain how quantitative variables were handled in the analyses. If applicable, describe which groupings were chosen and why | Figure 1 | SS, AS, PS, A-P Position, AIC, PIC, LAC, MAC, HF, WF,AEH, AEI were used as continuous variables.  750 non-TMD participants were selected and divided into three major groups (250 participants each): (1) openbite group (OBG) (2) crossbite group (CBG), and (3) control group (CG), according to the criteria.  Based on the characteristics of the TMJ’s growth and development, each major group was further divided by chronological age into five subgroups (50 participants each): groups Ⅰ (8–11 years), Ⅱ (12–15 years), Ⅲ (16–19 years), Ⅳ (20–24 years), and Ⅴ (25–30 years). |
| Statistical methods | 12 | (*a*) Describe all statistical methods, including those used to control for confounding | 10,11 | The normality of data was confirmed using the Kolmogorov-Smirnov test, and all data were normally distributed. Quantitative data are presented as mean ± standard deviation (SD).  A paired t-test was performed to compare the left and right sides of the TMJ. The trend between age and TMJ characteristics was assessed using a linear trend test (P for trend). For intergroup comparisons, one-way analysis of variance (ANOVA) and Tukey’s post hoc test were used to determine statistically significant differences among various groups.A two-way multivariate ANOVA (two-way MANOVA) was performed to estimate the composite effect of age and occlusal characteristics of anterior teeth on the TMJ.Statistical significance was set at P＜0.05. |
|  |  | (b) Describe any methods used to examine subgroups and interactions | 11 | Tukey’s post hoc test were used to determine statistically significant differences among various groups. |
|  |  | (c) Explain how missing data were addressed | Not applicable |  |
|  |  | (d)*Cohort study*—If applicable, explain how loss to follow-up was addressed  *Case-control study*—If applicable, explain how matching of cases and controls was addressed *Cross-sectional study*—If applicable, describe analytical methods taking account of sampling | Not applicable |  |
|  |  | (e) Describe any sensitivity analyses | Not applicable |  |
| **Results** |  |  |  |  |
| Participants | 13* | (a) Report numbers of individuals at each stage of study – eg numbers potentially eligible, examined for eligibility, confirmed eligible, included in the study, completing follow-up, and analysed | 7 | Following the application of inclusion and exclusion criteria, 750 non-TMD participants were selected and divided into three major groups (250 participants each): (1) openbite group (OBG) (81 males and 169 females; mean age: 18.18 ± 6.17 years), (2) crossbite group (CBG) (124 males and 126 females; mean age: 18.06 ± 6.90 years), and (3) control group (CG) (92 males and 158 females; mean age: 17.89 ± 5.66 years). |
|  |  | (b) Give reasons for non-participation at each stage | Not applicable |  |
|  |  | (c) Consider use of a flow diagram | Not applicable |  |
| Descriptive data | 14* | (a) Give characteristics of study participants (eg, demographic, clinical, social) and information on exposure and potential confounders. | Table 2, 3 |  |
|  |  | (b) Indicate number of participants with missing data for each variable of interest | Not applicable |  |
|  |  | (c) *Cohort stud*y-Summarise follow-up time (eg, average and total amount) | Not applicable |  |
| Outcome data* | 15* | *Cohort study*—Report numbers of outcome events or summary measures over time | Not applicable |  |
|  |  | *Case-control study—*Report numbers in each exposure category, or summary measures of exposure | Not applicable |  |
|  |  | *Cross-sectional study—*Report numbers of outcome events or summary measures | Table 2, 3, 4, 5 |  |
| Main results | 16 | (a) Give unadjusted estimates and, if applicable, confounder-adjusted estimates and their precision (eg, 95% confidence interval). Make clear which continuous variables were categorized. | Table 2, 3, 4, 5 |  |
|  |  | (b) Report category boundaries when continuous variables were categorized | Table2, 3, 4, 5 | 750 non-TMD participants were selected and divided into three major groups (250 participants each): (1) openbite group (OBG) (2) crossbite group (CBG), and (3) control group (CG), according to the criteria.  Each major group was further divided by chronological age into five subgroups (50 participants each): groups Ⅰ (8–11 years), Ⅱ (12–15 years), Ⅲ (16–19 years), Ⅳ (20–24 years), and Ⅴ (25–30 years). |
|  |  | (c) If relevant, consider translating estimates of relative risk into absolute risk for a meaningful time period. | Not applicable |  |
| Other analyses | 17 | Report other analyses done—eg analyses of subgroups and interactions, and sensitivity analyses | Table 4, 5 Figure 2 |  |
| **Discussion** |  |  |  |  |
| Key results | 18 | Summarise key results with reference to study objective | 19-24 | Condyles were positioned more posteriorly with increasing age in all groups, and the condylar position was more posterior in the OBG than in the CBG. The articular eminence inclination increased with age in all the groups. There were significant differences in the articular eminence inclination among the three major groups at the age of >15 years, and the condylar path was flatter in the CBG than in the OBG. |
| Limitations | 19 | Discuss limitations of the study, taking into account sources of potential bias or imprecision. Discuss  both direction and magnitude of any potential bias | 24 | This study has some limitations. Longitudinal data could better explain the relationship between the shape and position of the TMJ and incisor guidance than multi-cross-sectional data. |
| Interpretation | 20 | Give a cautious overall interpretation of results considering objectives, limitations, multiplicity of analyses, results from similar studies, and other relevant evidence | 23,24 | Functions and morphology are closely linked. It is widely recognized that pressure originating from the contractility of the masticatory muscles during chewing movements can affect the TMJ. This implies that the position and morphology of the TMJ are somewhat determined by the forces pressing on it. Various occlusal conditions result in functional adaptation in the neuromuscular system that is guided by proprioceptive feedback reflexes that originate in the teeth. This adaptation in turn influences the jaw muscles and causes structural and positional changes in the TMJ as a result of functional loads imposed on it. |
| Generalisability | 21 | Discuss the generalisability (external validity) of the study results | 24,25 | These findings can be clinically useful when evaluating the position and morphology of TMJ with CBCT images in individuals with anterior openbite and crossbite. |
| **Other information** |  |  |  |  |
| Funding | 22 | Give the source of funding and the role of the funders for the present study and, if applicable, for the  original study on which the present article is based | 26 | This study was supported by the National Science Foundation for Young Scientists of China [Grant No. 31800818] and the Program for the Natural Science Foundation of Chongqing, China [cstc2020jcyj-msxmX0191]. |

*Give information separately for exposed and unexposed groups.

Note: An Explanation and Elaboration article discusses each checklist item and gives methodological background and published examples of transparent reporting. The STROBE checklist is best used in conjunction with this article (freely available on the Web sites of PLoS Medicine at http://www.plosmedicine.org/, Annals of Internal Medicine at http://www.annals.org/, and Epidemiology at http://www.epidem.com/). Information on the STROBE Initiative is available at www.strobe-statement.org.
